# A Research Publication and Grant Preparation Program for Native American Faculty in STEM: Implementation of the Six R’s Indigenous Framework

**DOI:** 10.3389/fpsyg.2021.734290

**Published:** 2022-02-11

**Authors:** Anne D. Grant, Katherine Swan, Ke Wu, Ruth Plenty Sweetgrass-She Kills, Salena Hill, Amy Kinch

**Affiliations:** ^1^Mathematics, University of Montana, Missoula, MT, United States; ^2^Office of Research and Creative Scholarship, University of Montana, Missoula, MT, United States; ^3^Native American Studies, Nueta Hidatsa Sahnish College, New Town, ND, United States; ^4^Student Affairs, University of Montana, Missoula, MT, United States; ^5^Office of Organizational Learning and Development, University of Montana, Missoula, MT, United States

**Keywords:** indigenous research methodologies, professional development (PD), tribal college and university, institution of higher education, culturally responsive

## Abstract

Faculty members in science, technology, engineering, and mathematics (STEM) disciplines are typically expected to pursue grant funding and publish to support their research or teaching agendas. Providing effective professional development programs on grant preparation and management and on research publications is crucial. This study shares the design and implementation of such a program for Native STEM faculty (NAF-STEM) from two tribal colleges and one public, non-tribal, Ph.D. granting institution during a 3-year period. The overall development and implementation of the program is centered on the six R’s Indigenous framework – Respect, Relationship, Representation, Relevance, Responsibility, and Reciprocity. The role of NAF-STEM and their interactions with the program, as members of the community formed by their participation, impacted the program. Their practices and the program co-emerged over time, each providing structure and meaning for the other. Through such reciprocity, NAF-STEM and the program research team continually refined the program through their mutual engagement. They took on the shared responsibility of the program while they participated in and shaped its practices. The process and results of formative and summative assessment and the impact of COVID-19 on the program are reported. Results of the program offer lessons on the implementation of six R’s framework in professional development at institutions of higher education.

## Introduction

The need for a culturally responsive and effective professional development (PD) program to support Native American Faculty in Science, Technology, Engineering, and Mathematics (NAF-STEM) was identified through research into the experiences of Native American students in the field of natural resources and the critical contributions of Native American faculty to the success of Native American students (e.g., Aragon, 2002; Tippeconnic Fox, 2008; Gervais et al., 2016; Page-Reeves et al., 2018). A team assembled to create, implement, and study a model to support the career satisfaction and success of NAF-STEM, and to advance knowledge about issues impacting their career progression in STEM fields. Two Tribal Colleges and Universities (TCUs) and one predominantly white institution (PWI) with Native American and non-Native team members, formed the Willow Alliance, funded by the National Science Foundation.

The research team consists of 20 researchers. Ten of the team members are enrolled members of seven Tribal Nations; two are Asian and eight are non-Native. Four members of the team led the development and implementation of the Research Publication and Grant Preparation (RPGP) Program and are the first authors of this article. Three are Native, one is Asian. Between the two coauthors, one is Native, the other is White. The personal and professional lived experiences of the Native American team members contributed an additional layer of richness and perspective to the Willow project.

One of the project founders is a member of the Hidatsa tribe, who are also known as People of the Willows because historically, they lived along the river where willows were abundant. As the project was conceptualized, the vision of NAF-STEM as being similar to willows developed: a group of people who are thriving and play a critical role in their ecosystem. Willows represent flexibility and adaptation – not only to survive, but to thrive in some of the most challenging conditions and environments. The branches symbolize structure and a sense of responsibility. The roots symbolize being grounded and nurturing. The leaves symbolize nature and growth. Native Americans also use willows as a traditional medicine and willows are widely utilized in natural resources restoration for stream stabilization. The vision of the project was to create a model that supports NAF-STEM to become like the willows: abundant, contributing to a more diverse and enriched ecosystem, and a medicine for our people.

The research team developed a Willow model with three interconnected components to support NAF-STEM: Indigenous mentoring program (branches), institutional support program (roots), and research publication and grant preparation program (RPGP, leaves). The creation process of the model was Native American-led and was guided by specific tenets of Indigenous research methodologies (IRM), drawing upon Respect, Relationship, Representation, Relevance, Responsibility, and Reciprocity, our six R’s framework. In this article, we share the work on the RPGP component of the Willow model.

The definition of American Indian and Alaska Native varies across United States federal agencies and at different times in history. In this article, we use Native American, American Indian, American Indian and Alaska Native, Native, and Indigenous interchangeably. We are aware of the variation among the 500+ tribal nations in the United States and respect the differences in their traditions, cultures, languages, and worldviews. Here, we seek to look at Native American faculty broadly, focusing on commonalities among these groups.

### A Brief Description on the History and Contexts of Tribal Colleges and Universities

The first Tribal College was established by the Navajo Nation in 1969 to provide culturally sensitive, place-based higher education to Native Americans. As of the American Indian Higher Education Consortium [AIHEC] (2021) reports there are 37 Tribal Colleges and Universities (TCUs) in the United States, spanning 16 states and providing rigorous education to predominantly Native American students. Chartered by their respective Tribal councils, TCUs tend to be community hubs centered on the economic and cultural needs of their students (St. Pierre, 1998; Page, 2017).

Tribal Colleges and Universities are classified separately from other institutions of higher education, which fall under several familiar categories, such as Doctoral Universities and Baccalaureate Colleges (Indiana University Center for Postsecondary Research, 2021). According to an Introduction to Tribal Colleges from AIHEC (1999), most TCUs have small student populations; most are remotely located on reservations with limited access to other colleges; all began as 2-year colleges, and all have open admission policies. AIHEC also indicates that most TCUs are teaching institutions and do not offer tenure or have an instructional ranking system. The student body at TCUs consists primarily of Native American (about 89%) students (cited by Voorhees, 2003, using IPEDS Fall 2000 Enrollment Survey) with enrollment typically ranging from a few hundred to a couple thousand students.

### Demographics of Native Faculty at Tribal Colleges and Universities and Non-Tribal Colleges and Universities

In 2018, in higher education institutions nationwide, less than 1% of faculty were Native American (Institute of Education Sciences, U.S. Department of Education, and National Center for Education Statistics (NCES), 2020). Among TCUs in 2014, 33% of faculty were Native American, 82% had a Master’s degree or higher, and 68% were full-time (Al-Asfour, 2014). TCUs draw strength from their reliance on cultural scholars to lead courses centered on the delivery of cultural knowledge and/or language. Thus 11% of faculty, staff, and administrators are listed as experts in their field with no degree (AIHEC and Systemic Research, Inc., 2008).

A 2008 AIHEC report indicates that many faculty members at TCUs commit a high level of effort to student support services and few faculty receive release time, which means they have less time to develop research products (e.g., publications, books, presentations).

### Role of Native American Faculty at Tribal Colleges and Universities and Non-Tribal Colleges and Universities

Tribal Colleges and University faculty are paid less than faculty at PWIs (average $18,000 less), but TCU faculty, especially American Indian faculty, have a strong sense of obligation and commitment to Native communities (Voorhees, 2004). Further, Native American faculty at TCUs share many core values with their Native counterparts at non-TCUs, including a desire to *give back* to their community (Page-Reeves et al., 2019). In a 2014 study, Yeager found that racial minorities tend to persist at higher rates when they have a more “self-transcendent” view of tedious academic activities (Yeager et al., 2014). Many Native Americans hold such a view, being motivated by family and a strong sense of giving back (Guillory, 2008; Guillory and Wolverton, 2008). This self-transcendent view often goes a step further – to a sense of duty to their families and communities (Al-Asfour, 2014; Page-Reeves et al., 2019).

Pursuing grant funding is a common expectation of faculty in STEM disciplines. Providing effective PD programs on grant preparation and management can help advance their careers. However, institutional contexts and culture are important factors that support or constrain faculty research activities (Zimbler, 2001). In this study, the Willow PD program aimed to support NAF-STEM at two TCUs and one public Ph.D. granting PWI, taking into account the participants’ needs and their institutions.

## The Six R’s Indigenous Framework

The overall design of the RPGP is centered on the six R’s framework for Indigenous research: Respect, Relationship, Representation, Relevance, Responsibility, and Reciprocity. The ideology behind the six R’s has been put into practice in Indigenous communities and elsewhere for generations. They came into the practice fairly recently and not all at the same time.

Three decades ago, Verna J. Kirkness and Ray Barnhardt laid the groundwork stressing the need to incorporate into higher education systems, The Four R’s: Respect, Relevance, Reciprocity, and Responsibility (1991). The authors presented American Indian students’ perspectives that differed from mainstream institutions and characterized ways programming transforms education (Thorne, 2019). Over time, the fifth R for relationship came into play (Harris and Wasilewski, 2004; Wilson, 2008; Styres and Zinga, 2013; Cull et al., 2014; Tessaro et al., 2018).

Tessaro et al. (2018) expounded upon “The Five R’s for *Indigenizing* Online Learning,” examining how a Canadian First Nations course for school principals was centered around the Five R’s. Representation was the sixth R to be included. Representation of Indigenous communities has been a struggle since colonization, and the ability to “represent ourselves” is seen as a fundamental right (Smith, 2012). Kovach (2010) stresses the importance of including Indigenous voice and representation within research, using conversation as a means for gathering knowledge through the relational process of story-telling.

Stemming from Indigenous worldviews, the six R’s honor Indigenous knowledge systems and support cultural integrity. Below we describe the six R’s in more detail. They do not stand alone, they complement one another. They are connected, intertwined, and overlap. The six R’s are core values woven throughout our work that together provide a holistic structure guiding this study.

**Relationship** requires attention and effort to build and maintain (Brayboy and Maughan, 2009; Brayboy et al., 2012). *“Relationship is the kinship obligation”* (Cajete, 2000; Harris and Wasilewski, 2004). Respect, relevance, reciprocity, and responsibility are expressed through relationship (Brayboy et al., 2012; Styres and Zinga, 2013). Relationship is reciprocal and respectful (Kirkness and Barnhardt, 1991). We recognize building trust and good relationships with our participants is fundamental.

**Respect** is recognition of a community’s cultural standards and openness to learning (Carjuzaa and Fenimore-Smith, 2010). We recognize and respect the “mutually empowering” aspect of the relationship between individuals and the group (Hampton, 1988; Kirkness and Barnhardt, 1991). Taking the time and making the time to build relationships demonstrates respect for Indigenous values and the community as a whole (Brayboy et al., 2012; Windchief et al., 2017). Truly respecting our participants involves learning who they are: their identities, culture, values, and stage in their professions, all of which impact how we develop, shape, and change our PD program.

**Responsibility** is all-inclusive, recognizing our connections to Indigenous communities and our desire to continually develop sustainable, supportive relationships with them (Cull et al., 2014). “*Responsibility is the community obligation”* (Harris and Wasilewski, 2004). It is our responsibility to our participants to develop this program to support their professional career progression; their institutions (e.g., when faculty grow and succeed, their students and the institution also grow and benefit from each other); their/our communities; and to support their individual understanding of and definition/s of success. Rather than developing the program FOR participants, it is our responsibility to co-create the PD program WITH participants.

**Representation** allows the community to identify what is relevant. The participants’ unique knowledge traditions are represented in new contexts through their participation. The Willow team allows representation of Native participants and provides space to have their voices heard. Our NAF-STEM participants’ input supports the direction of the PD program.

**Relevance** values Indigenous knowledge, involves Indigenous communities, and ensures that programs, services, and education for Indigenous peoples are responsive to the needs they *themselves* have identified (Cull et al., 2014). We ensure that our PD is relevant to our participants’ individual and institutional contexts and goals. Inclusion of their voices and insights make the research and program relevant.

**Reciprocity** is respectful knowledge sharing between people participating as both student and teacher, across disciplines, throughout the full educational process (Brayboy et al., 2012; Cull et al., 2014). *“Reciprocity is the cyclical obligation”* (Harris and Wasilewski, 2004). Reciprocity plays a critical role in participants’ lives and “unifying cultural construct” (Guillory, 2008; Page-Reeves et al., 2019). It is important that the PD program is mutually beneficial to our participants and the program team. We learn from each other and continuously co-construct the program together.

## Professional Development Program – Research Publication and Grant Preparation

### Background of the Professional Development Participants

The RPGP had eight NAF-STEM participants (four female, four male). Their home institutions are in the Northern Great Plains. Four participants are from two different TCUs and four participants are from one state-funded 4-year PWI.

### Overview of Research Publication and Grant Preparation

**FIGURE 1:**
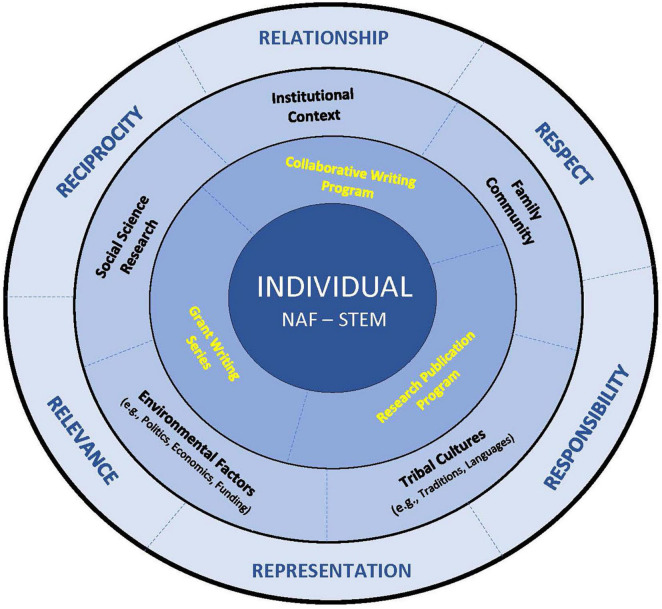
Six R’s indigenous framework for RPGP.

Figure 1 illustrates how the six R’s serve as the overarching framework, which surround our work and encompass all other elements of the RPGP. From inclusion to integration of Indigenous perspectives and approaches, NAF-STEM are *represented* at the center of our continued journey.

The circles illustrate our ongoing relationship building with each other, our communities, our environments and beyond. Through our collaboration, we recognize and understand that our shared knowledge unites us, providing a cohesive Indigenous voice for PD in higher education, and elevating our Indigenous communities and institutions. In this realm, we are able to shift institutional approaches away from *merely tolerating* Indigenous knowledge(s) “to one where Indigenous knowledge(s) are embraced as part of the institutional fabric” (Pidgeon, 2016).

The RPGP was designed as a 1-year program that offered three components to Willow NAF-STEM participants (highlighted in yellow text in Figure 1). They could participate in one or more components of the program.

Component 1: A grant proposal preparation program. If a participant chose to join this component, the expected outcome was that, by the end of the 1-year program, the participant would complete a review-ready proposal for an external funding source as a PI or a collaborative proposal as PI or Co-PI.

We offered two mechanisms to support participants to achieve this goal: a Grant Writing Series (GWS) and Collaborative Writing and Support (CWS). The GWS offered four, 90-min sessions to collaboratively explore different aspects of writing grant proposals. All sessions were in person and online. The CWS was based on a best practice in grant writing, i.e., to have periodic reserved writing time with peers for accountability and support (Young et al., 2016). Participants had the option of meeting together as a large group or in subgroups for the 90–120 min CWS sessions where they received support from the team.

Component 2: A research publication program. The expected outcome was that the participant would complete a submit-ready, peer-reviewed journal article, conference proceeding, or book chapter with the participant serving as a major contributor (e.g., lead, second, or third author). NAF-STEM who chose this option participated in at least two of the four GWS sessions in option 1. Each individual worked with the research team to determine the time for regular Collaborative Writing and Support (CWS) based on availability, location, and format. The research team worked with individual participants at the frequency they wanted.

Component 3: A collaborative writing program (CWP) among participants on their experiences as Native faculty and researchers. The expected outcome was a submit-ready manuscript co-authored by participants for a journal or alternative destination determined by authors. The Willow team facilitated regular CWS meetings for participants who chose to work on the manuscript.

### Calendar Schedule of the Research Publication and Grant Preparation

Table 1 demonstrates the timeline and tasks of the RPGP. Due to the COVID-19 pandemic, the development and implementation of the program lasted over 3 years.

**TABLE 1:** Timeline of the RPGP program.

Task	Timeline
Develop materials for GWS and relationship building	Summer (Year 1)
Pilot the GWS	Fall semester (Year 1, in person among faculty at the non-TCU institution)
Adapt the materials for the GWS	Spring semester (Year 1)
GWS 1	May (Year 2, in person at the same non-TCU institution and online)
GWS 2, GWS 3	Summer Workshop (Year 2, in person at one of the partner TCUs and online)
GWS 4	September (Year 2, in person at the non-TCU and online)
CWS (weekly)	October through June (Year 2, held weekly in person and online); A subgroup of the CWS extended their meetings into Year 3 due to COVID-19 pandemic and all meetings were online
Reflection	Summer Workshop (Years 3 and 4, originally planned at the other partner TCU, but was moved online due to COVID-19 pandemic)

### Implementation of Research Publication and Grant Preparation

**FIGURE 2:**
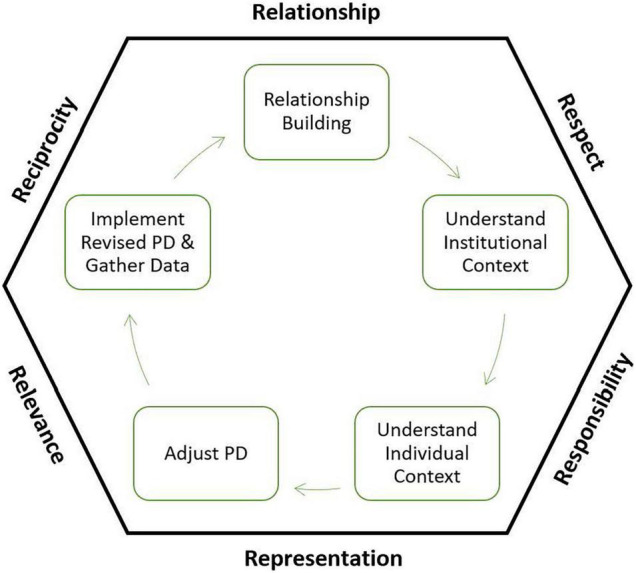
Five key elements of RPG.

Figure 2 demonstrates the five key elements of the development process of RPGP under the guidance of six R’s. The circular connection (green arrows in Figure 2) indicates the interactive and iterative nature among all the elements.

The five elements from Figure 2 are described below. Additional detail on how the six R’s were necessary for program adaptation is described in the section “Discussion” of this article.

#### Relationship Building With Fellows

Relationships were fostered through face-to-face interactions at group sessions, one-on-one correspondence (phone, email, Slack messages), and through an open-door policy. Ongoing interactions helped our team to further understand participants’ individual contexts in relation to proposal writing and grant management.

#### Understanding Institutional Context

Tribal Colleges and University context and participants’ needs differ from public state-funded institutions. TCUs tend to be very student-focused and encourage their faculty to embody this in their day-to-day work. At many public state-funded institutions, faculty are expected to perform research and are sometimes afforded resources (e.g., time, proposal writing assistance, databases) to accommodate this. This difference means that the number and types of proposals that a faculty member submits depends upon their institutional context.

#### Understanding Individual Context

Participants had various degrees of grant writing and management experience. Those with more experience became peer mentors for others. This informed our decision to adapt the program to engage the senior faculty in discussions during the GWS sessions.

Other “intersectional” traits for participants included tribal cultures and traditions, family composition and status (e.g., single parent, foster parent), individual professional status, and environmental factors such as a global pandemic.

#### Adjusting Professional Development

The understanding of individual context led us to realize that some participants prefer to publish their scholarly work, rather than securing grants due to institutional and individual factors. Many faculty in our program opted to focus on publishing their work to strengthen their research agenda. This resulted in further adaptation of our PD program by adding Component 2 – Research Publications.

Through our reciprocal relationships and respectful listening, we learned that several participants desired to share their lived experiences as Native faculty in TCU and non-TCU settings to (a) support Native communities to inspire more students into STEM fields and become faculty and (b) help the broader audience in higher education to better understand the strengths/challenges for Native STEM faculty and provide suggestions on support. This resulted in adding Component 3 – the Collaborative Writing Program.

#### Implementing Revised Professional Development and Gathering Data

Over the period of the project, we revised the initial grant writing and management program to better meet the needs of our participants. We implemented our professional development plan, gathered data, and made adjustments (as described above). This process resulted in the final program, where participants were allowed to choose the component(s) that best suited their personal and professional goals: Component 1: Grant Writing Series; Component 2: Research Publications; Component 3: Collaborative Writing Program.

### Program Assessment

The program effectiveness was assessed through formative and summative assessments. The formative assessments included: participation rates in the program and conversations between external evaluators and participants at biannual gatherings and summer retreats for feedback.

Summative assessments include: (a) final outcomes from the RPGP, that is, number of proposals submitted and awarded for Component 1; number of publications submitted and accepted for publication for Component 2; number of publications or presentations for Component 3; (b) findings from informal focus group with participants at the end of the program.

## Results

In this section we present the formative and summative assessment results of the RPGP. We then share the results on the program effectiveness from our participants’ perspective. We also address how the COVID-19 pandemic impacted the RPGP and our response to it.

### Results From Program Assessment

Results from Component 1 GWS: Among the eight NAF-STEM, the numbers of participants in GWS sessions 1–4 are 3, 8, 8, and 5 (note that participants only need to attend two of the four GWS sessions). One of the TCU NAF-STEM participants chose to further develop and submit a proposal in collaboration with a faculty member at the state institution to start a new graduate program at the TCU. The proposal was selected for funding.

Results from Component 2 Research Publication: Two NAF-STEM chose to participate in this component working at their own pace. Based on one participant’s writing log, he used 15 writing sessions, ranging from 1 to 5 h long, to work on a manuscript. The manuscript has been published in a refereed journal in his field. The other participant published one book chapter.

Results from Component 3 CWP: Five participants collaborated with three research team members on this component. This component was extended from a 1-year program to a 20-month period due to the COVID-19 pandemic. The group had a total of 38 1-h gatherings to work on the writing project. The participants gave a presentation followed by question-and-answers with about 50 participants at a national conference for Native American and Chicano/Hispanic students. A manuscript authored by the four participants and three team members on how Native American faculty navigate academia is currently being revised for its second submission to a peer review journal.

### Results on Program Effectiveness Through the Lens of the Participants

Regarding the effectiveness of the program, we learned that participants appreciated the opportunity to get to know one another and share their stories. They felt that the work in RPGP was meaningful and gave them the ability to reclaim knowledge in an Indigenous context. These conversations also allowed Indigenous knowledge to expand beyond the TCUs and into other institutions, where the conversation on cultural change was brought to a wider audience. Others felt that the program could have been improved by offering a less time-consuming option. Some participants with ample grant writing, management, and research publication experiences indicated that they did not learn new skills through the program, and the RPGP could have benefited from an even deeper contextual understanding of where participants are, both in terms of career trajectory and institution type.

We also asked participants to posit what a similar program might look like if it were implemented solely at a TCU or solely at a PWI. One participant indicated that many TCUs do not ask that their faculty write proposals, but rather hire grant writers to support institutional-level proposals. There is no pressure from the institution for the NAF-STEM to pursue individual research funding. Taking this comment into account, we might avoid proposal writing for PD at TCUs altogether, potentially replacing it with advice for seeking or working with a professional grant writer (depending on the needs of the individual TCU faculty). Because NAF tend to have a self-transcendent approach to their work, broadening the definition of knowledge production beyond research publications and into creative scholarship would be beneficial. Including creative dissemination products (e.g., documentary, film, podcasts) would be useful in a classroom and in the community, therefore reciprocating the NAF’s impact beyond their own research and career and into their community.

### Impact of COVID-19 Pandemic

The COVID-19 pandemic challenged the implementation of the RPGP by limiting interaction among participants. The collaborative writing sessions and annual workshops were shifted from in-person to abbreviated remote formats, limiting interactions and reducing opportunities that could potentially lead to publication.

Additionally, Native American (NA) communities experienced the pandemic in especially devastating ways. In the United States the COVID-19 mortality rate was 2.5 times higher among NA than it was among non-Hispanic whites (Akee and Reber, 2021). These high rates were also reflected in the states where our faculty participants’ home institutions are located. For instance, in Montana, NAs account for roughly 7% of the population, but accounted for 32% of COVID-19 deaths (Montana Department of Public Health and Human Services [DPHHS], 2020-2021). The drastic disparity in the COVID-19 death rate added strain to our NA participants, several of whom experienced personal loss during the pandemic, as well as impacts to productivity that are impossible to measure.

We responded with flexible timelines to complete the RPGP and added a weekly “wellness check-in” for our team and NAF-STEM to provide support to each other. We purchased technology and provided financial support to alleviate some of the added pressure on participants.

## Discussion

In this section, we discuss the findings of the study through the conceptual lens of six R’s. We conclude with reflections on lessons learned and recommendations for researchers and administrators in institutions of higher education.

### The Strengths of Incorporating the Six R’s Indigenous Framework

Incorporating the six R’s (Relationship, Respect, Responsibility, Reciprocity, Representation, and Relevance) into the RPGP served two purposes. When working in systems that value non-Indigenous processes, it was important for the Willow team to *remain grounded* in the Indigenous six R’s framework. Using the six R’s framework also provided the Willow team with a *system of accountability* aligned with Indigenous practices.

Many of the ways the six R’s guided the process were complex and integrated. In this section, we discuss the development and implementation of the RPGP and the holistic strengths of the six R’s, which are congruent with Indigenous Research Methodologies (IRM).

The connections and overlapping of the six R’s is important. Highlighting how they are represented and connected to each other in the Willow project is integral to understanding the process. The first of the six R’s recognized in this process is **Representation. Representation** existed at the beginning, with its development by several Native American team members leading the RPGP and elevating Native perspectives. NAF shared narratives throughout, contributing to important national conversations on using IRM in science communities with their shared work. Having a shared identity with NAF-STEM, Willow team members as a whole carried a sense of **Responsibility** with **Reciprocity** in RPGP development. This sense of **Responsibility** is represented by Willow team leader’s responsiveness to NAF-STEM needs, while simultaneously NAF-STEM reciprocated responsiveness to the needs of their campus community. The responsiveness of the modifications are connected to **Relationship, Respect,** and **Reciprocity**.

The Willow team facilitators entered into **Relationship**s with NAF-STEM using a **Respect**ful **Reciprocal** approach. As participants began to express needs and interests to modify the structure of the RPGP, the Willow team **Respect**ed their requests to make modifications. Because of the **Respect** given to NAF-STEM, they were open to **Reciprocate** and express what their hopes were for the program.

As a result, a new option, the Collaborative Writing Project (CWP), was developed and took on a different, less hierarchical structure. All participants, whether they were Willow team or NAF-STEM, held equal influence. In this collective approach, as NAF-STEM felt compelled to direct the conversations, Willow rotated leadership of meetings and sections.

As the CWP was developed, NAF-STEM identified **Relevant** needs at each of their institutions. They were given opportunities to tell their stories – stories about themselves, their students, their experiences, and their communities. This meaningful and sustainable engagement with the Indigenous community is **Relevance** (Cull et al., 2014; Stanton et al., 2019). **Responsibility** contributed to the Willow team’s support in adjusting to this new structure that would meet the needs of their respective campuses. **Reciprocity** was practiced in the knowledge-sharing that happened among the group and in the value of meeting the needs of NAF-STEM, who in turn felt compelled to give back to their students, campuses and communities.

A strong sense of community was formed and close **Relationships** were made among Willow participants with implementation of the six R’s. The participants’ shared identity allowed an openness to be responsive and flexible, share their work with each other and the wider audience, and learn about each other. Shared identity and experiences enhanced focus on culture and language.

Building **Relationship**s with TCUs and communities is crucial to better understanding Native scholars’ perspectives and interest in program components that support student involvement and are meaningful to the TCU community. The opportunity to successfully co-develop and implement the RPGP with NAF-STEM was possible because of the six R’s framework.

### Limitations, Lessons Learned and Reflections

The development of a model by this project has not been done with the intention of creating a copy-paste program that can be replicated across institution types and populations. As the program evolved, it became apparent that the standard goal of a repeatable PD was not going to be one of our outcomes. Our results demonstrate the critical importance of assessing and engaging participants in the development stages and program delivery to better meet their unique needs. Similar to traditional knowledge systems, NAF-STEM needs are unique and context-dependent, which makes exact replication illogical and undesirable, especially for dissimilar populations. A model approach would be thoughtful engagement and respectful listening for participants to identify strategies and supports best suited to their specific needs and desires – with implementation of the six R’s throughout.

To appreciate the TCUs NAF-STEM’s efforts in our program, we originally planned to pay for a course teaching release, so that they would have time to participate. We quickly realized that our TCU partner institutions are geographically located in rural areas and the number of faculty in STEM is very small. It is extremely difficult to hire qualified instructors to teach their courses. Willow changed the compensation plan to summer salary, travel funds, and seed funding.

We are grateful that our NAF at the TCUs participated in our program in ADDITION to their heavy teaching loads and service requirements. Building a trustworthy relationship with participants takes time and it cannot be done through a one-time survey or meeting. A wide variety of flexible communication options with NAF-STEM is needed to suit individual preferences. PD activities must be carefully planned and continually adapted to the unique and individual needs and responsibilities of participants.

### Contributions to Professional Development Field

The RPGP expands on existing models of PD. For example, J. M. Frantz’s approach to providing research and writing support for a group of health professionals used “academics’ needs as a departure point for designing activities that support them throughout the process” (Frantz, 2012, p. 122). Bali and Caines (2018) describe faculty programs based on transformative learning and heutagogy that respect individuals’ priorities, reward PD, promote self reflection, and support access (through technology in their case). RPGP was reconfigured to address faculty priorities, offered a stipend for participation, provided both time and topics that allowed for self reflection and used various formats. It met all of Bali and Caines’ goals while also introducing the six R’s framework, critical to making it relevant to NAF-STEM. Our work of incorporating current best practices in the context of the six R’s contributes to the field of participatory PD with a specific lens on Indigenous scholars.

### Conclusion

This article shared the iterative development and implementation of the RPGP, a PD program to support NAF-STEM at two TCUs and one PWI. The RPGP offered a set of evolving options for participants that allowed for professional outcomes, such as a grant proposal, book chapter, and article submissions, as well as contributions to participants’ communities through a TCU graduate program proposal, a presentation, and an article on NAF navigating academia. Future iterations could have an even broader definition of professional products and could reduce or remove the grant writing components for participants from TCUs. Feedback from participants emphasized that the RPGP allowed them to reclaim knowledge in an Indigenous context.

The six R’s Indigenous framework (Respect, Relationship, Representation, Relevance, Responsibility, and Reciprocity) guided the RPGP team to emphasize Native perspectives, respond to participants’ needs and contexts, and support participants’ desires to give back to their communities. Native communities have had the experiences of western researchers and large institutions conducting research on Native communities with unethical approaches and without truly building long-lasting, reciprocal relationships or understanding the contexts of Indigenous cultures, traditions, needs, and ideologies. We hope this example of adaptive program development helps researchers better understand the importance of learning and applying the six R’s Indigenous framework when working with tribal communities and Native peoples.

## Data Availability Statement

The original contributions presented in the study are included in the article/supplementary material, further inquiries can be directed to the corresponding author.

## Ethics Statement

The studies involving human participants were reviewed and approved by the IRB at University of Montana, Salish Kootenai College, and Sitting Bull College. The patients/participants provided their written informed consent to participate in this study.

## Author Contributions

All authors listed have made a substantial, direct, and intellectual contribution to the work, and approved it for publication.

## Conflict of Interest

The authors declare that the research was conducted in the absence of any commercial or financial relationships that could be construed as a potential conflict of interest.

## Publisher’s Note

All claims expressed in this article are solely those of the authors and do not necessarily represent those of their affiliated organizations, or those of the publisher, the editors and the reviewers. Any product that may be evaluated in this article, or claim that may be made by its manufacturer, is not guaranteed or endorsed by the publisher.
